# Initial Relative Position Influencing Self-Assembly of a Black Phosphorus Ribbon on a CNT

**DOI:** 10.3390/ijms19124085

**Published:** 2018-12-17

**Authors:** Jing Cao, Yixuan Wang, Jiao Shi, Junrui Chai, Kun Cai

**Affiliations:** 1 State Key Laboratory of Eco-hydraulics in Northwest Arid Region of China, Xi’an University of Technology, Xi’an 710048, China; caojingxn@163.com (J.C.); wangyixuan940520@163.com (Y.W.); jrchai@xaut.edu.cn (J.C.); 2 State Key Laboratory of Geo-Information Engineering, Xi’an 710048, China; 3 Key Laboratory of Agricultural Soil and Water Engineering in Arid and Semiarid Areas, Ministry of Education, Northwest Agriculture and Forestry University, Yangling 712100, China; 4 State Key Laboratory of Structural Analysis for Industrial Equipment, Dalian University of Technology, Dalian 116024, China; 5 Centre for Innovative Structures and Materials, School of Engineering, Royal Melbourne Institue of Technology University, Melbourne 3001, Australia

**Keywords:** black phosphorus, carbon nanotube, self-assembly, molecular dynamics

## Abstract

It is difficult to obtain a nanotube from phosphorus with a 3*sp*^2^ electron configuration by chemical synthesis. However, a physical fabrication approach, such as self-assembly, is worth trying. In an experiment, when using a carbon nanotube (CNT) to trigger self-assembly of a black phosphorus (BP) ribbon, the final configuration of the BP component may be sensitive to the initial relative position of the CNT to the BP ribbon. For instance, using the same CNT with different initial relative positions to the BP ribbon, the BP ribbon may finally become a nanotube, or a scroll, or just wind upon the CNT, or escape from the CNT, etc. In this study, the sensitivity is investigated using molecular dynamics simulations. Numerical results illustrate some essentials for potential fabrication of a BP nanotube from ribbon.

## 1. Introduction

A two-dimensional crystal is a planar crystal of nanometre thickness stacked by several single atomic layers. Due to its unique electrical, optical, and magnetic properties, the two-dimensional crystal, such as graphene [1], silicene [2], molybdenum disulphide [3], or black phosphorus (BP) [4,5,6], has attracted extensive attention for wide applications. In a few-layered BP, van der Waals (vdW) interactions exist between neighbouring layers [7,8]. Each internal phosphorus atom in the single-layered BP is covalently bonded (3*sp*^3^) with three neighbouring phosphorus atoms. The electron configurations of the atoms in BP induce excellent electric properties [9,10,11,12], e.g., direct band gap, high electron mobility. For example, field-effect transistors based on BP crystals with high charge-carrier mobility were verified in experiments [10]. 

Similar to carbon nanotubes (CNTs) being formed from curved graphene ribbon, a BP nanotube [13,14,15,16,17,18,19,20,21,22,23,24,25,26] may also be obtained by curling a single-layer black phosphorus ribbon and bonding the closing neighbour edges. One of the merits of the new phosphorus allotrope is that the tube has fewer unsaturated atoms, which are essential to the thermal and chemical stabilities of the material [27]. Theoretically, the thermal and mechanical properties of the one-dimensional phosphorus nanotube from a single-layered BP have been investigated recently [17,18,19,20,21,22,28,29]. For example, results [19] indicate that the thermal stability of a curved BP ribbon depends on the bending direction of the ribbon. The BP ribbon curved along the zigzag direction is brittle because the P–P bonds can bear small tensile strain. Buckling behaviour of the BP nanotube or with the protection from a CNT under axial compression was tested [20,21]. Zhao et al. [30] studied the stability of a chiral BP nanotube under uniaxial compression and compared the results with those of commensurate BP nanotubes. Shi et al. [22] evaluated the stability of a BP nanotube covered by a carbon nanotube under centrifugal force. Liu et al. [31,32] investigated the strength of stability of BP nanotubes with defects. Rouhi et al. [33] studied the vibration property of armchair BP nanotubes using finite element models, whose parameters were given by density functional theory.

However, before application, BP nanotubes should be fabricated or synthetized. One fact that the P–P bond is slightly stronger than the vdW interaction between the neighbour phosphorus atoms in different layers makes the chemical synthetizing method of BP nanotubes full of challenges. Hence, the physical assembly approach could be worth trying. To obtain a BP nanotube, Cai et al. [23,24,25] tested several approaches to produce a nanotube from a rectangular BP ribbon. They [23] found that to form a perfect BP nanotube on a specified CNT, a rectangular BP ribbon with a perfect length is required. Otherwise, a BP scroll will be obtained. The other method is to use a CNT bundle to trigger the self-assembly of a rectangular BP ribbon into a nanotube by moving one or more carbon nanotubes [24]. Fullerenes were also adopted to drive the self-assembly of a BP ribbon into a tube [25]. In their work, an ideal BP nanotube can be obtained at extremely low temperature. Using CNTs with larger radii, an ideal BP nanotube can be formed at higher temperature. Sometimes, the self-assembly process can be easily controlled at higher temperature [26].

In the above study, all the CNTs and the BP ribbons have similar relative positions. However, in an experiment, when using a CNT to trigger the self-assembly of a BP ribbon, the initially relative position of the CNT has great influence on the self-assembly process, but is hard to control. The reason is that the initially relative position between the CNT and the BP ribbon determines the attraction distribution on the BP atoms and further influences the motion/deformation of the ribbon. To reveal the detailed effect of the relative position on the self-assembly process of the BP ribbon, in this study, we put a CNT nearby a parallelogram BP ribbon with different distances and different angles. Models and methodology are introduced in Section 2. Numerical results are given in Section 3 with the discussion. In Section 4, some conclusions are drawn for potential applications.

## 2. Numerical Results and Discussion

### 2.1. Winding of a BP Ribbon on a CNT with α = 0°

**Figure 1:**
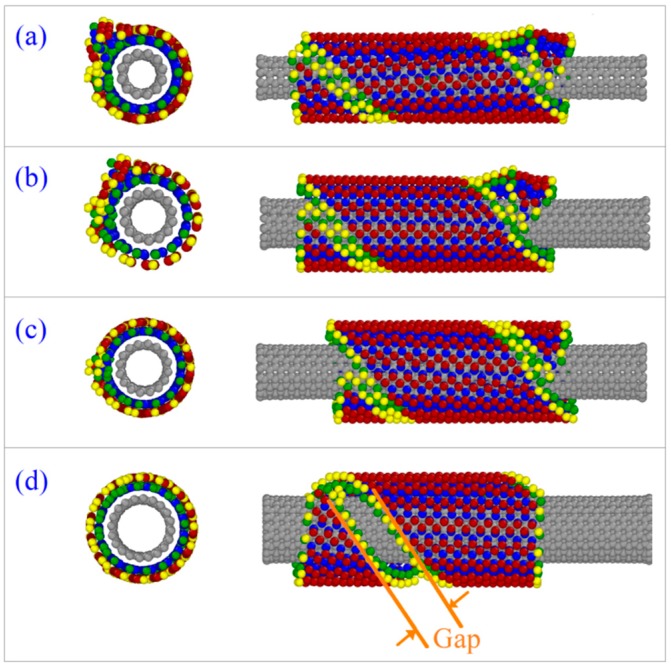
Snapshots of the system at 500 ps when α = 0°, *L*y = 0 at 14 K. (**a**) On CNT (6, 6). (**b**) On CNT (7, 7). (**c**) On CNT (8, 8). (**d**) On CNT (10, 10).

Table 1 and Figure 1c indicate that when the CNT axis is parallel to the *z*-axis, i.e., α = 0°, the BP ribbon can form into an ideal nanotube only upon the CNT (8, 8) when *L*y = 0 nm. On the CNTs (6, 6) and (7, 7) used, the BP nanotubes formed from the ribbon have defects at the tube ends (Figure 1a,b). If the CNT (10, 10) is adopted, the BP ribbon can only wind upon the CNT, but fails to form into a nanotube. The reason is that the gap between the two oblique edges of the curly BP ribbon is too high and attraction between them is too weak to let the two edges move closer to each other for bonding (Figure 1d).

**Figure 2:**
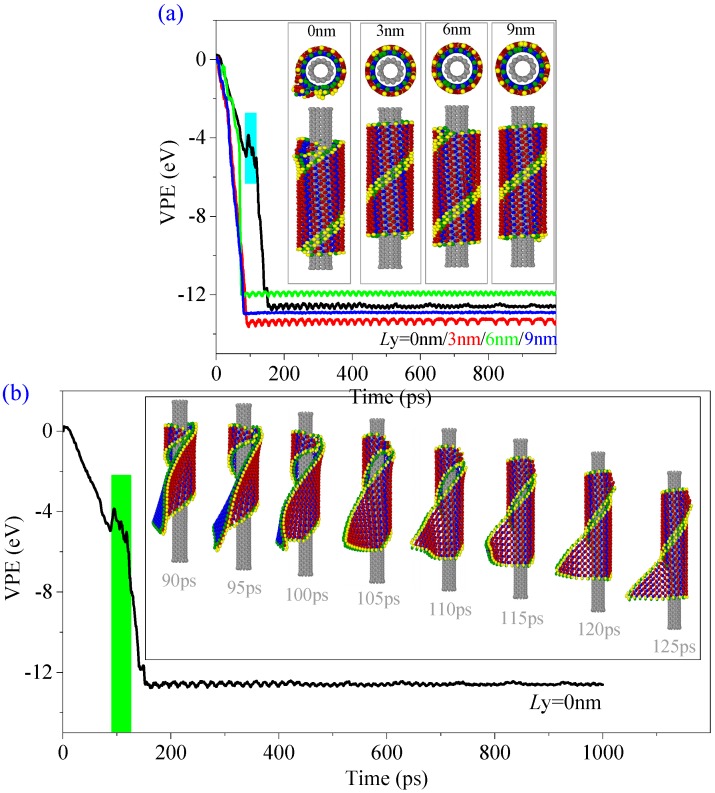
Historical curves of the variation of potential energy (VPE) of the BP during winding upon the CNT (6, 6) with α = 0° at 14 K. (**a**) VPE curves of the BP with different values of *L*y. Snapshots of the final state of the BP ribbon are inserted. (**b**) Winding process of the BP ribbon on the CNT with *L*y = 0 nm.

When the CNT moves right, i.e., *L*y > 0 nm, more atoms on the BP ribbon are closer to the CNT, and the final configurations of the four chiral BP nanotubes after assembly were different. The BP nanotube can be formed on both the CNTs (6, 6) and (7, 7) (inserts in Figure 2). However, the BP ribbon still cannot form into a tube on the CNT (10, 10). Hence, we conclude that the value of *L*y is not the essential factor when the BP ribbon can only wind upon a CNT with a larger radius (Table 1).

**Figure 3:**
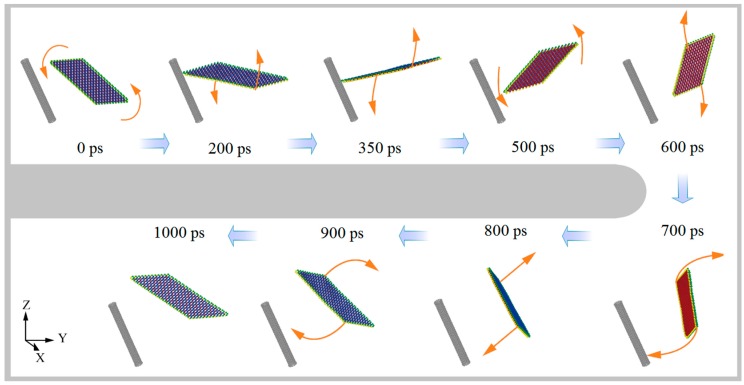
Some representative system configurations of “escape” with CNT (6, 6) when α = 30° and *L*y = 0.

Winding of BP ribbon on CNTs with α = ±30°: The effect of correlation between the parameters, i.e., *L*y and α, for determining the positions of the CNTs is necessarily demonstrated. As listed in Table 2, for the CNTs with rotational angle α = 30°, they cannot capture the BP ribbon due to negligible attraction when *L*y ≤ 3 nm. In this condition, the ribbon escapes rather than winds upon the CNTs (Figure 3). This is because the distance between the CNTs and the BP ribbon is too high and attraction upon the BP ribbon is too weak. Without getting closer to the CNTs, the ribbon has no chance to be curved and further forms into a tube. If *L*y = 6 or 9 nm, the distance between the two components is less than 1 nm (the cut-off of the L–J potential), and the BP ribbon can wind upon the CNTs to form the BP nanotube.

**Figure 4:**
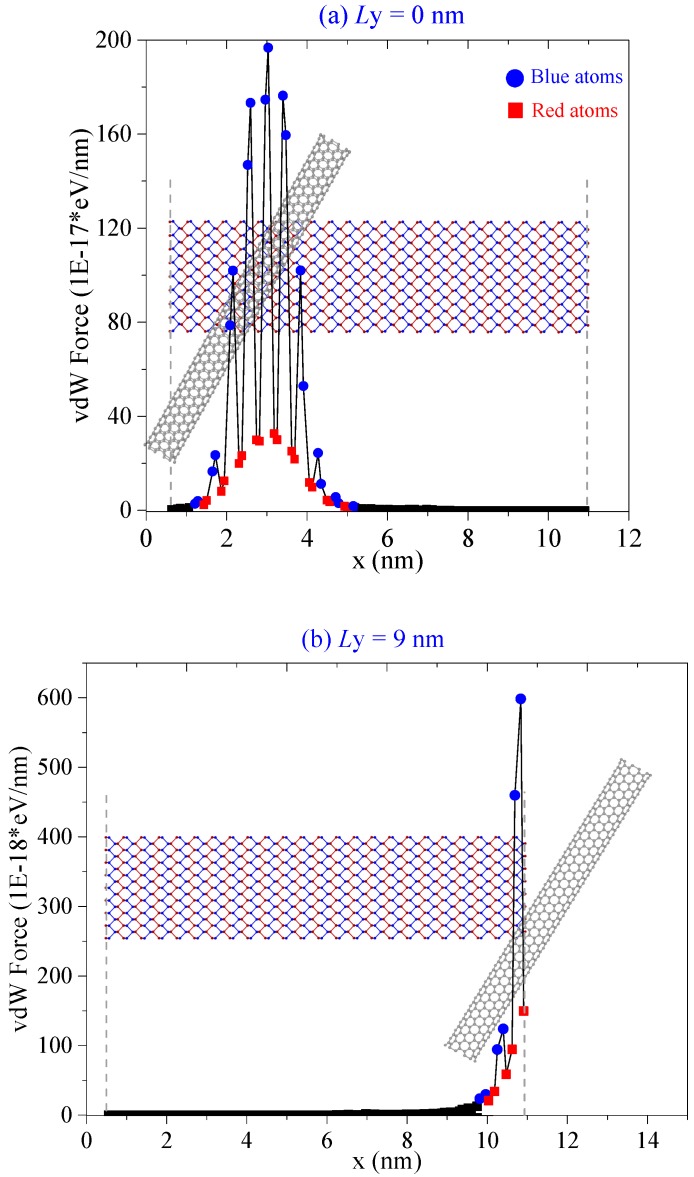
Resultant van der Waals force of the CNT (10, 10) on each column of phosphorus atoms in the BP ribbon with α = −30°. (**a**) *L*y = 0 nm. (**b**) *L*y = 9 nm.

If α < 0°, e.g., −30°, the CNTs can attract the BP ribbon effectively even when *L*y = 0 nm. The ribbon does not escape any more. It can form into a tube with or without a defect, or forms into a scroll, or just winds upon a CNT with higher radius (Table 3). For example, at *L*y = 6 nm, the distance between the CNTs and the BP ribbon reaches the minimum among the four cases. Only in this case, the ribbon becomes an ideal nanotube regardless of the CNTs’ radii. Therefore, the translation of CNT along the y-direction influences the final configuration of BP because of different initial distributions of attractive force on the BP ribbon (Figure 4). 

**Figure 5:**
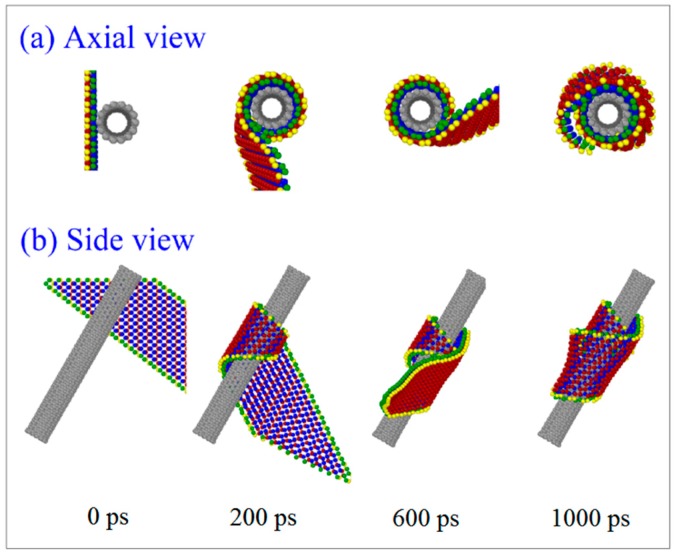
Snapshots in the winding process of BP ribbons with the CNT (7, 7) system with *L*y = 0 nm, α = −30°. (**a**) Axial view. (**b**) Side view.

**Figure 6:**
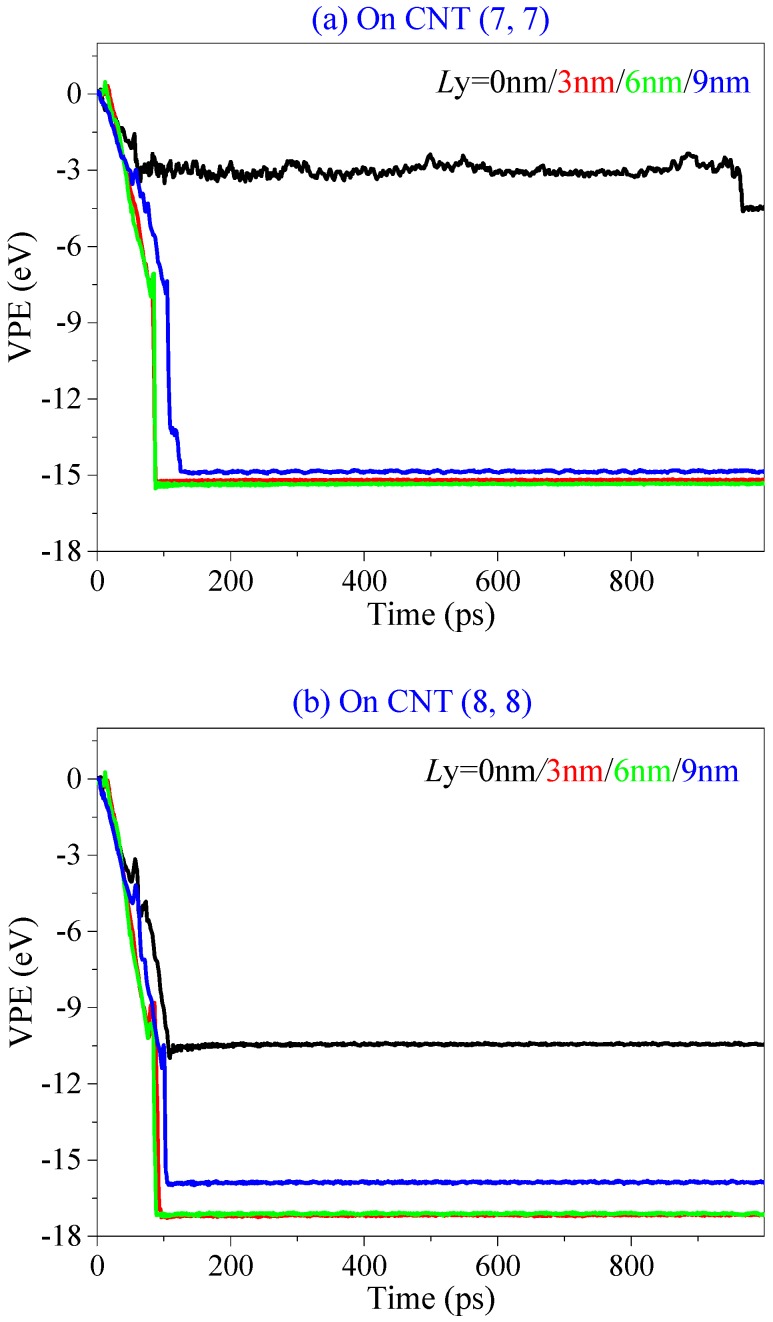
Historical curves of VPE of the BP during winding upon CNT with α = −30°, but different values of *L*y. (**a**) On CNT (7, 7). (**b**) On CNT (8, 8).

In Table 3, the BP ribbon forms into a nanoscroll on the CNT (7, 7) when *L*y = 0 nm. Figure 5 gives the representative snapshots to indicate the assembly process (Movie S1 in Supplementary Materials). It can be found that the ribbon first winds upon the CNT (7, 7) and then winds upon itself (snapshot at 1000 ps in Figure 5). The two oblique edges overlap rather than bond together well. According to the rest of the VPE curves of the system with respect to the CNTs (7, 7) (Figure 6a) and (8, 8) (Figure 6b), the system with *L*y > 0 nm becomes stable after no more than 180 ps, the ribbon forms into a tube, simultaneously. Hence, the state of the system after 200 ps does not change obviously. The sudden drop in each VPE curve tells the history of bonding between the two oblique edges on the BP ribbon.

**Figure 7:**
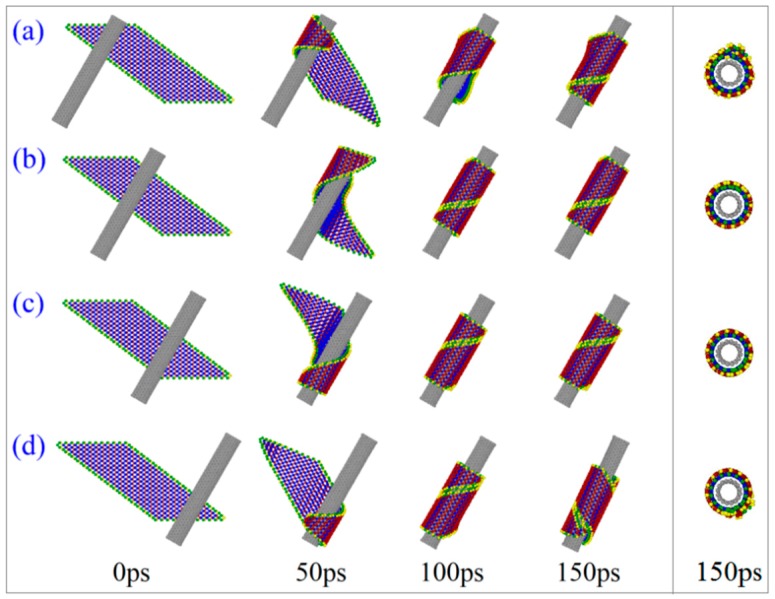
Snapshots in winding process of BP ribbons nearby the CNT (8, 8) with α = −30°, but different values of *L*y. (**a**) *L*y = 0 nm. (**b**) *L*y = 3 nm. (**c**) *L*y = 6 nm. (**d**) *L*y = 9 nm.

When moving the oblique CNT (8, 8) from left to right (*L*y = 0, 3, 6, 9 nm), the self-assembly processes of the ribbon are illustrated by the snapshots of the BP during winding upon CNT between 0 and 150 ps, as shown in Figure 7. The BP ribbon starts to curve at different locations (Loc), where most atoms are attracted by the CNT (e.g., Figure 4). For example, when the CNT is near the top left corner of the BP ribbon (*L*y = 0 nm), the BP begins to curve at this location due to the local strong attraction from the CNT. When *L*y = 9 nm, the BP nanotube has a defect after winding upon the CNT. Perfect BP nanotubes are only formed on the CNT with *L*y = 3 and 6 nm (Figure 7b,c). 

### 2.2. Effect of α on Self-Assembly of the BP Ribbon

**Figure 8:**
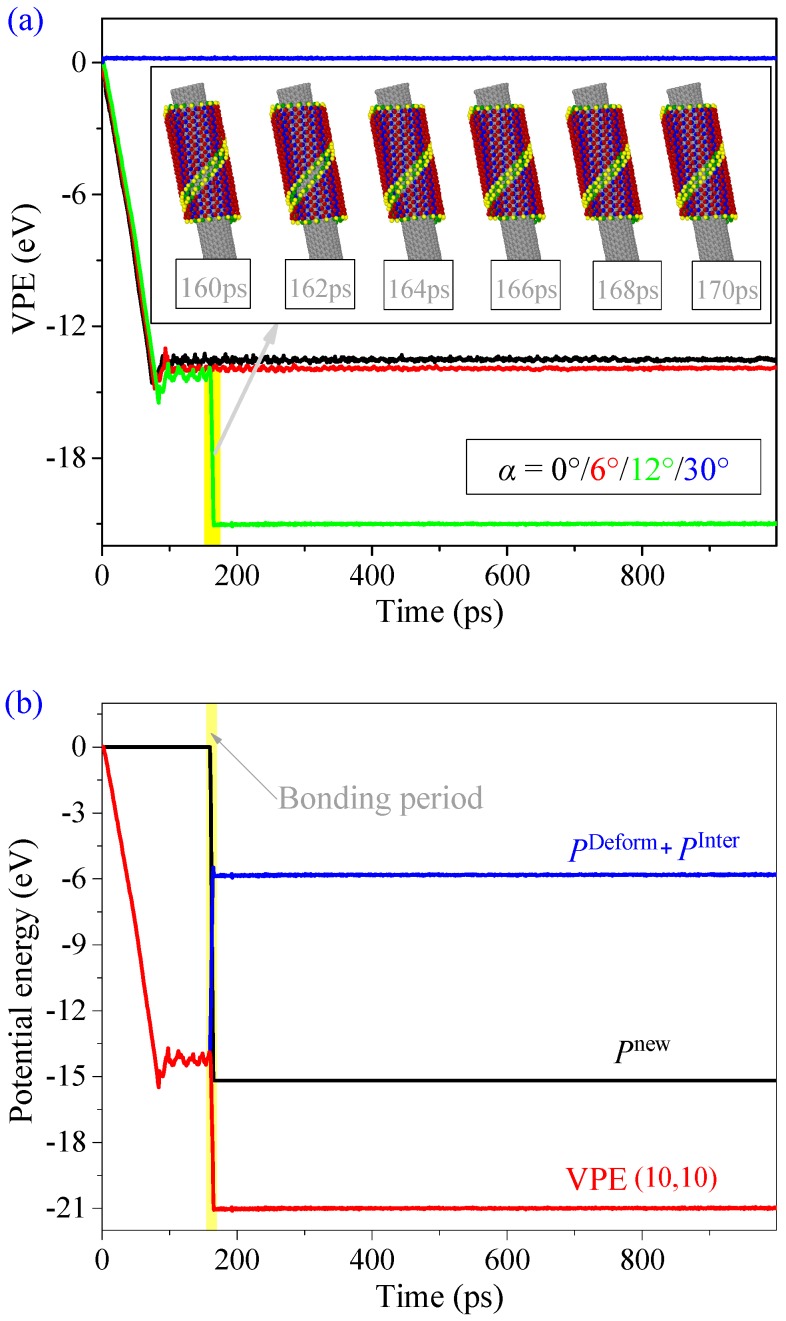
Historical curves of the potential energy of the system and snapshots of the BP on the CNT (10, 10) when *L*y = 3 nm. (**a**) VPE of the system with different α. (**b**) Combination of VPE with α = 12°.

To illustrate the influence of CNT’s rotation angle α on the self-assembly of the BP ribbon, the CNT (10, 10), which has a large radius, is first considered in simulations with the results listed in Table 4. The ribbon’s behaviour depends on the initial conditions. For example, when *L*y = 0 nm, the BP ribbon escapes directly from the CNT if α > 3°. When *L*y = 3 nm (Table 4), the state of the BP component as a different experience. For example, firstly, in the case of α = 0° and 3°, the BP ribbon just winds upon the CNT, but cannot form into a nanotube (Wind only). Secondly, for the cases of α = 6° and 9°, the state of the BP ribbon is between “Wind only” and “Tube”, i.e., “Wind only
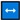
Tube” (Movie S2 in Supplementary Materials). In this case, the distance between the two oblique edges is smaller than that in “Wind only” (Figure 1d), but still slightly longer than the bond length of P–P. Thirdly, for the CNT with α between 12° and 18°, it can trigger a successful self-assembly of the ribbon into a nanotube. When the ribbon is attracted and starts to wind on the CNT, the P atoms on the oblique edges move closer to each other and finally bond together (Figure 8a,b). Finally, when the rotation angle is more than 30°, the distance between BP and CNT becomes higher, and the BP ribbon escapes due to the lack of attraction. The final configuration of the BP structure is sensitive to the value of α due to the zigzag potential barriers on the oblique edges of the BP ribbon.

Dividing the VPE of BP in the system with *L*y = 3 nm and α = 12° into two parts, i.e., new P–P bonds induced VPE, and the remaining part due to deformation together with the interaction between the two components (Figure 8b), we find that the value of *P*^New^ starts decreasing at 162 ps and keeps unchanged after 166 ps. During the bonding period, 23 new P–P bonds are generated between the two helical edges of the ribbon. In the same period, the value of *P*^Deform^ + *P*^Inter^ jumps up. This is mainly caused by the deformation of the BP component from ribbon to tube.

As *L*y becomes higher (e.g., 6 or 9 nm), the BP ribbon has difficulty “escaping” from the CNT (Table 4). If it does not escape, the ribbon can become a nanotube at a higher value of α, or between the “Wind only” and “Tube” at a lower value of α. However, the angle interval for forming a BP nanotube is difficult to obtain because random vibration of atoms on the ribbon may lead to failure of tube formation.

**Figure 9:**
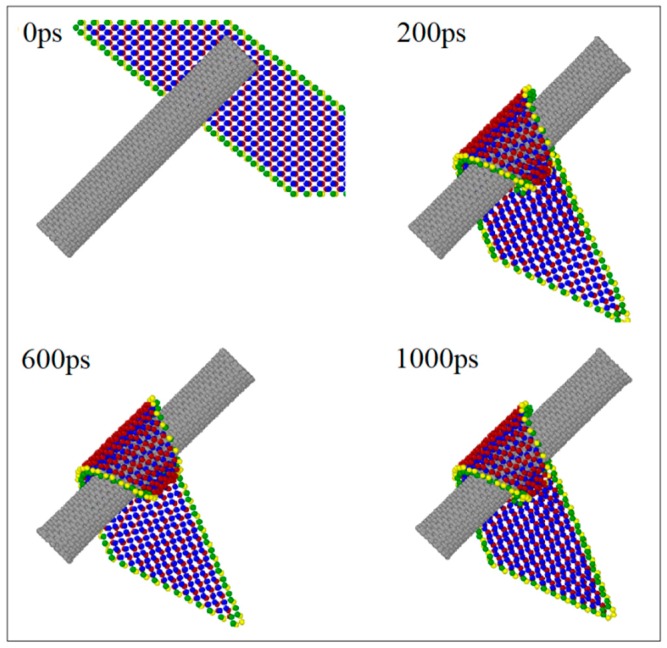
Snapshots in winding process of the BP ribbon on CNT (10, 10) with *L*y = 0 nm and α = −45°.

What would happen to the BP component if α < 0°? As α is negative, more atoms on the BP ribbon are closer to the CNT. If the CNT with *L*y = 0 nm can drive the self-assembly of the BP ribbon, so it does at *L*y > 0 nm. Hence, the self-assembly process of the ribbon on the CNTs with *L*y = 0 nm is considered, and the results are listed in Table 5. The table indicates that the formation of a BP nanotube depends both on the value of α and the radius of CNT. For instance, the BP ribbon on the CNT (6, 6) could form into an ideal nanotube when α ≤ −9°; otherwise, the BP tube has a defect. On the CNT (7, 7), α ≤ −15° should be satisfied to form into a tube or a scroll. At a smaller angle of the CNT, the BP ribbon can easily form into a nanotube on the CNT (8, 8), but has difficulty at a larger angle of α. For example, the BP ribbon may become a nanotube with a defect or a scroll. If the CNT (10, 10) is used, the ribbon has difficulty becoming a nanotube. In most cases, the ribbon just winds upon the CNT. At a higher angle, the ribbon may form into a scroll or even half of the ribbon wind upon the tube; the remaining part does not curve (“1/2 scroll” in Figure 9, Movie S3 in the Supplementary Materials). 

## 3. Models and Methods

### 3.1. Models

**Figure 10:**
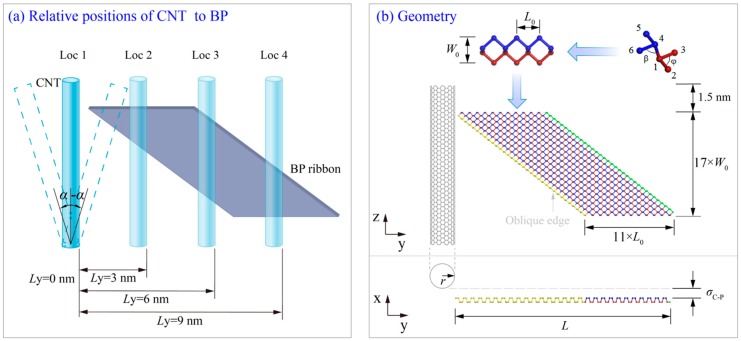
The system of a carbon nanotube (CNT) and a parallelogram black phosphorus (BP) ribbon. (**a**) Relative position of the structure. (**b**) Configuration of the system with *L*y = 0.

The model shown in Figure 10 contains a CNT and a parallelogram black phosphorus nanoribbon. The relative position of the CNT is determined by the value of *L*y and α. The average C–C bond length is found to be 0.142 nm. Lattice parameters *l*_14_ = 0.2244 nm, *l*_12_ = *l*_13_ = *l*_45_ = *l*_46_ = 0.2224 nm; angles β = 102.09°, φ = 96.36°, *L*_0_ = 0.44 nm, and *W*_0_ = 0.33 nm. x-distance σ_C__–__P_ = ~0.34 nm. The length of the BP ribbon along the y-direction is *L* = 28*L*_0_ = ~12.8 nm. The axial length of CNT is 8.682 nm. The ends of the CNTs are hydrogenated in order to improve the stability of the edge carbon atoms on CNTs and to avoid bonding interaction between the CNT and the BP ribbon. Within 0.5 nm of each hydrogenated end of CNT, the atoms are fixed in simulation. In the model, different CNTs with different relative positions will be considered. Details of the parameters of models are listed in Table 6. To illustrate the relative position effect on the self-assembly process, we set up different initial positions for the carbon nanotube. The angle with the z-direction (α) and position along the y-direction of the CNT (*L*y) are two factors considered in the simulation as shown in Figure 10, i.e., 

Factor 1: α = 0°, ±3°, ±6°, ±9°, ±12°, ±15°, ±18°, ±30°, ±45°, ±60°;

Factor 2: *L*y = 0, 3, 6, and 9 nm.

### 3.2. Methods

#### 3.2.1. Methodology

In this work, molecular dynamics simulations are fulfilled via the open source code LAMMPS to show the response of the BP ribbon nearby a CNT [34]. In a simulation, empirical potentials are used to estimate the interactions among atoms. For instance, AIREBO potential is adopted to calculate the interaction between carbon and/or hydrogen atoms in the CNT. The strength of covalent bonds between neighbour phosphorus atoms in the BP ribbon is evaluated using the Stillinger–Weber potential [35], whose parameters were provided by Jiang [36]. The nonbonding interaction between two atoms is described using the Lennard–Jones (L–J) potential [37], i.e.,

(1) $$\Pi_{ij}^{LJ}=4\varepsilon_{ij}\left[{\left(\sigma_{ij}/r_{ij}\right)}^{12}-{\left(\sigma_{ij}/r_{ij}\right)}^{6}\right]$$

where $\Pi_{ij}^{LJ}$ is the potential energy between atom *i* and atom *j*, $\varepsilon_{ij}$ is the depth of the potential well, $\sigma_{ij}$ the distance between atoms *i* and *j* when the interaction potential between particles is zero, and *r_ij_* is the spatial distance between atom *i* and atom *j*. Parameters in the L–J potential for carbon, hydrogen, and phosphorus atoms are listed in Table 7.

The variation of potential energy (VPE) of the system is calculated and can be used to describe the variation of the system configuration. The value of VPE can be obtained by subtracting the initial potential energy of the component from the current potential energy, i.e.,

(2) $$\mathrm{VPE}\left(t\right)=P_{\mathrm{system}}\left(t\right)-P_{\mathrm{CNT}}\left(t_{0}\right)-P_{\mathrm{BP}}\left(t_{0}\right)=P_{C-P}^{\mathrm{Inter}}+P_{P-P}^{\mathrm{new}}+P_{\mathrm{CNT}}^{\mathrm{Deform}}+P_{\mathrm{BP}}^{\mathrm{Deform}}$$

where $P_{\mathrm{system}}\left(t\right)$ is the total potential of the system at time t; $P_{\mathrm{CNT}}\left(t_{0}\right)$ and $P_{\mathrm{BP}}\left(t_{0}\right)$ are the potential energies of the CNT and BP structures at $t_{0}$, respectively. $P_{C-P}^{\mathrm{Inter}}$ is the potential energy of vdW interactions between carbon and phosphorus atoms; $P_{P-P}^{\mathrm{new}}$ is the potential energy due to generating new P–P bonds (each bond results in ~0.66 eV of decreasing potential energy); $P_{\mathrm{CNT}}^{\mathrm{Deform}}$ and $P_{\mathrm{BP}}^{\mathrm{Deform}}$ are the potential energy induced by geometric deformation of the CNT and BP ribbon, respectively.

#### 3.2.2. Flowchart of an MD Simulation

**Figure 11:**
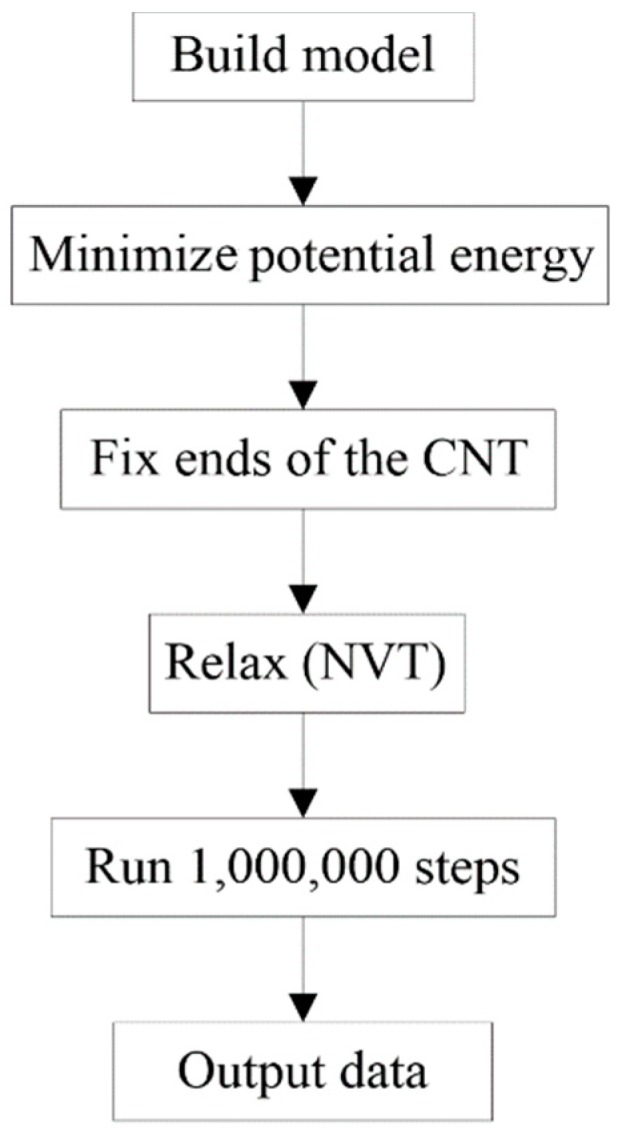
Major steps in an MD simulation.

Figure 11 shows the flowchart of MD calculation. Briefly, first, build a parallelogram BP ribbon and CNT with a specified initial relative position; second, reshape the system by minimization of the potential energy of the system; third, fix both ends of the CNT; fourth, put the system under a canonical (NVT) ensemble with the Nosé–Hoover thermostat to control temperature [38,39]; fifth, run 1,000,000 steps and record relevant data; the time step for the integral is set at 0.001 ps; finally, stop for post-processing. 

## 4. Conclusions

When using a CNT to trigger self-assembly of a BP ribbon, the initial relative positions of CNTs to the BP ribbon influence the final configuration of the BP structure. The final state of the BP component after self-assembly depends on the radius of the CNT, the initial location, and the rotational angle of the CNT. According to the molecular dynamics results and discussion, some conclusions can be drawn for potential fabrication of a BP nanotube by the self-assembly approach, i.e.,

(1) Using a CNT with α = −30° and *L*y = 0 nm, the BP ribbon can form into a tube, scroll, or just winds up the CNT. The final configuration depends on the diameter of the CNT.

(2) When putting the BP ribbon nearby the same CNT with the given value of *L*y, the final configuration of the BP structure depends on the value of α due to zigzag potential barriers on the oblique edges of the BP ribbon.

(3) When α > 0°, the BP ribbon can form into a tube on the CNT with a larger diameter when the ribbon starts winding upon the CNT from its lower-right corner. 

(4) When α < 0°, the BP ribbon can easily form into a tube on a slimmer CNT with *L*y = 0.

## Figures and Tables

**Table 1:** Final states of the BP with 4 types of CNTs at different initial positions along the y-direction with α = 0° at 14 K. “Tube” means the BP ribbon forms into an ideal tube. “Defect” means the BP nanotube has a defect. “Wind only” represents the ribbon just winds upon the CNT, but does not form into a tube.

Position	On (6, 6)	On (7, 7)	On (8, 8)	On (10, 10)
*L*y = 0 nm	Defect	Defect	Tube	Wind only
*L*y = 3 nm	Tube	Tube	Tube	Wind only
*L*y = 6 nm	Tube	Tube	Tube	Wind only
*L*y = 9 nm	Tube	Tube	Tube	Wind only

**Table 2:** Final states of the BP nearby different CNTs at different values of *L*y, but the same α = 30°. “Escape” means the tube cannot capture the BP ribbon.

Position	On (6, 6)	On (7, 7)	On (8, 8)	On (10, 10)
*L*y = 0 nm	Escape	Escape	Escape	Escape
*L*y = 3 nm	Escape	Escape	Escape	Escape
*L*y = 6 nm	Tube	Tube	Tube	Tube
*L*y = 9 nm	Tube	Tube	Tube	Tube

**Table 3:** Final states of the BP on different CNTs with α = −30°, but different initial positions. “Scroll” means the BP ribbon winds upon the CNT and forms into a scroll.

Position	On (6, 6)	On (7, 7)	On (8, 8)	On (10, 10)
*L*y = 0 nm	Tube	Scroll	Defect	Tube
*L*y = 3 nm	Tube	Tube	Tube	Wind only
*L*y = 6 nm	Tube	Tube	Tube	Tube
*L*y = 9 nm	Tube	Tube	Defect	Tube

**Table 4:** Final states of the BP structures with CNT (10, 10) at different angle (α > 0°) with the *z*-direction.

Angle of CNT	*L*y = 0 nm	*L*y = 3 nm	*L*y = 6 nm	*L*y = 9 nm
α = 0°	Wind only	Wind only	Wind only	Wind only
α = 3°	Wind only	Wind only	Tube	Wind only
α = 6°	Escape	Wind only<–>Tube	Wind only<–>Tube	Wind only<–>Tube
α = 9°	Escape	Wind only<–>Tube	Wind only<–>Tube	Tube
α = 12°	Escape	Tube	Wind only<–>Tube	Wind only
α = 15°	Escape	Tube	Tube	Tube
α = 18°	Escape	Tube	Wind only	Tube
α = 30°	Escape	Escape	Tube	Tube
α = 45°	Escape	Escape	Escape	Tube
α = 60°	Escape	Escape	Escape	Escape

**Table 5:** Final states of the BP component on CNTs when α < 0° and *L*y = 0 nm.

Angle of CNT	On (6, 6)	On (7, 7)	On (8, 8)	On (10, 10)
α = 0°	Defect	Defect	Tube	Wind only
α = −3°	Defect	Defect	Tube	Tube
α = −6°	Defect	Defect	Tube	Wind only
α = −9°	Tube	Defect	Tube	Wind only
α = −12°	Tube	Defect	Tube	Wind only
α = −15°	Tube	Tube	Tube	Wind only
α = −18°	Defect	Tube	Tube	Wind only
α = −30°	Tube	Scroll	Defect	Tube
α = −45°	Tube	Tube	Scroll	1/2 Scroll
α = −60°	Tube	Tube	Defect	Tube

**Table 6:** Parameters in different models.

Model	CNT (n, m)	Diameter of CNT (nm)	Length of CNT (nm)	Number of Atoms		
				Carbon	Phosphorus	Hydrogen
1	(6, 6)	0.804	8.679	840	904	24
2	(7, 7)	0.941	8.681	980	904	28
3	(8, 8)	1.081	8.682	1120	904	32
4	(10, 10)	1.356	8.682	1400	904	40

**Table 7:** The L–J potential parameters among carbon, hydrogen, and phosphorus atoms.

Atom *i*	Atom *j*	*σ_ij_* (nm)	*ε_ij_* (meV)
P	C	0.34225	6.878
P	P	0.34380	15.940
C	C	0.34000	2.844
C	H	0.30250	2.065
H	H	0.26500	1.499

## Supplementary Materials

Supplementary materials can be found at http://www.mdpi.com/1422-0067/19/12/4085/s1.
